# Promoting and hindering factors for implementation of the Infant Stool Colour Card in Dutch youth health care organizations

**DOI:** 10.1007/s00431-025-06212-7

**Published:** 2025-06-04

**Authors:** L. T. Warris, C. A. Dogger, S. A. Reijneveld, J. B. F. Hulscher

**Affiliations:** 1 Dept. of Health Sciences, University Medical Center Groningen, University of Groningen, Groningen, The Netherlands; 2 Netherlands School of Public & Occupational Health, Utrecht, The Netherlands; 3 GGD Regio Utrecht, Zeist, The Netherlands; 4 Dept. of Surgery, University Medical Center Groningen, University of Groningen, Groningen, The Netherlands

**Keywords:** Biliary atresia, Infant Stool Colour Card, Implementation, Screening, Youth health care

## Abstract

Biliary atresia is a rare life-threatening cholestatic disease of early infancy, and early treatment largely improves its prognosis. Currently, use of the Infant Stool Colour Card (ISCC) to improve early detection is implemented in several youth health care (YHC) organizations in the Netherlands. We aimed to map the promoting and hindering factors for implementation of the ISCC. We performed a qualitative study with semi-structured interviews among small and large Dutch YHC organizations. We included organizations that implemented the ISCC, and that did not. For both, we interviewed youth public health (YPH) physicians and nurses involved in the organizational policy regarding hyperbilirubinemia, using an interview topic guide based on the Consolidated Framework of Implementation Research. Ten of 17 approached YHC organizations participated (with 10 physicians and 3 nurses), among which all three organizations that implemented the ISCC. Major promoting factors were the positive attitudes of YPH professionals towards the ISCC, a sense of competence among YPH professionals, the ISCC’s simplicity, the compatibility with workflows, and the involvement of key stakeholders. Major hindering factors were limited knowledge and inadequate risk perception of biliary atresia among YPH professionals and general practitioners (GPs), insufficient ISCC integration within YHC organizations (lack of repeated training and lack of a designated responsible professional), and insufficient collaboration with GPs.

*Conclusion*: To implement use of the ISCC, comprehensive, interactive training and education on its rationale and use for YPH professionals and GPs are essential. The successful embedding of the ISCC into YHC organizations requires a designated professional.

**Table:** 

**What is Known:** • *There is a delay in diagnosing biliary atresia in the Netherlands.* • *The Infant Stool Colour Card led to earlier diagnosis and surgery in different countries, which largely improves the prognosis of biliary atresia.* **What is New:** • *The Infant Stool Colour Card is included in the new Dutch national guideline ‘Early detection and diagnosis of biliary atresia’ as a standard screening method in case of prolonged jaundice in youth health care organisations.* • *This study maps barriers and promoting factors of the ISCC to ensure successful implementation within Dutch youth health care organisations.*

**Supplementary Information:**

The online version contains supplementary material available at 10.1007/s00431-025-06212-7.

## Introduction

Biliary atresia is a rare cholestatic disease of early infancy. In the Netherlands, approximately 10 children are affected annually (1:19:000) [1]. Early symptoms consist of prolonged jaundice (≥ 2 postnatal weeks) and the development of pale-coloured stools. This life-threatening progressive disease is the most common indication for liver transplantation in children (~ 50%) [2]. Successful timely surgical restoration of the bile flow (Kasai portoenterostomy) can postpone and even prevent the need for liver transplantation [1, 3]. Therefore, early recognition and diagnosis of biliary atresia is crucial. Internationally, the quality criterium for early Kasai is before 60 days of life. In the Netherlands, only 56% of children with biliary atresia undergo surgery before this age (median age of surgery: 56 days, range 27–143) [4]. Delays in early detection are primarily due to the difficulty in distinguishing jaundice caused by biliary atresia from the more common, harmless breast milk jaundice [5]. Both conditions initially present in a similar way. Moreover, parents, youth public health (YPH) physicians, and general practitioners (GPs) often fail to recognize discoloured stool [5].

**Fig. 1:**
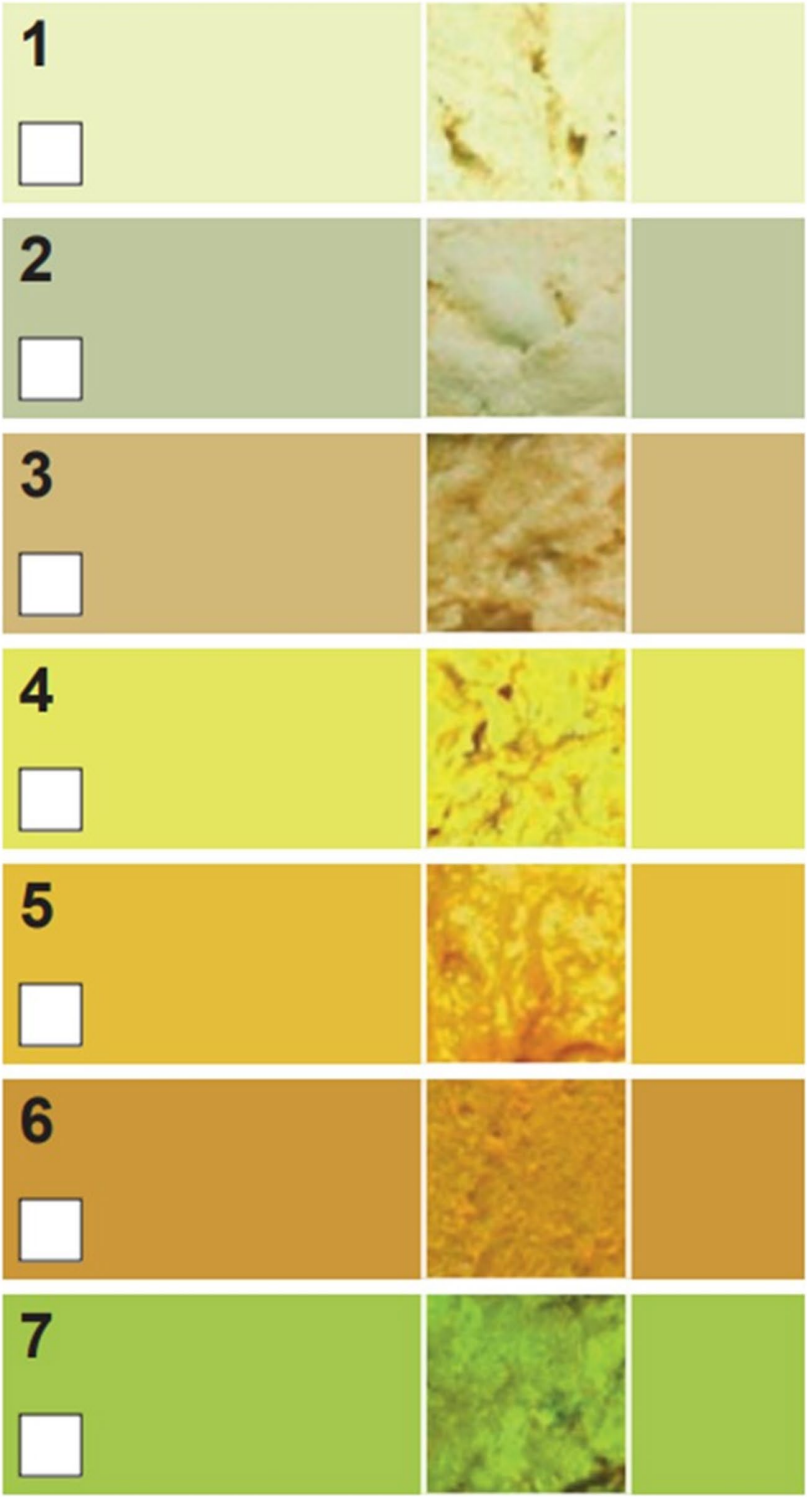
Infant Stool Colour Card. The colours 1–3 on this card can be considered abnormal

A national screening with the Infant Stool Colour Card (ISCC) could contribute to early recognition and operation. This simple and cheap screening method consists of seven photographs of different coloured stool samples from infants, both normal and abnormal (Fig. 1). A Canadian study (with similar biliary atresia incidence) demonstrated ISCC’s cost-effectiveness in universal screening for biliary atresia by passive card distribution [6]. Implementation costs easily outweigh the significant expense of a liver transplant after a failed Kasai (€100,000 in the first year alone). Studies in different countries [7–14] showed that ISCC implementation led to earlier diagnosis and surgery (Taiwan: 65.7% Kasai < 60 days instead of 49.5% Kasai < 60 days [7–10]), and an improved 5-year survival with the native liver (89.3% versus 55.7%, *P* < 0.001 [7–10]).

The Dutch multidisciplinary guideline for hyperbilirubinemia [15] recommends a bilirubin blood test for prolonged jaundice at 3 weeks of age. In the Netherlands, 95% of all children between 2 and 6 weeks of age visit YPH professionals at well-child clinics. In case of suspected biliary atresia, these professionals mostly have to refer infants to a general practitioner (GP) to order a bilirubin test, and to a paediatrician in case of conjugated bilirubin level > 10 umol/l or > 20% of total bilirubin.

Following a successful pilot program, three Dutch YHC organizations (covering approximately 13,000 births annually, amounting to 6% of the Dutch newborns) have implemented ISCC into their routines. The ISCC is presented to parents by nurses during home visits at 2 weeks of age. Discoloured stool identified through the ISCC prompts a referral to a paediatrician. To improve early detection rates nationally, the recently published Dutch national guideline ‘Early detection and diagnosis of biliary atresia’ [15] includes the ISCC as a standard screening method in addition to the bilirubin test in case of prolonged jaundice. For its successful implementation, evidence is needed on barriers and promoting factors. Therefore, this study aims to map the factors that promote and hinder the implementation of the ISCC as a screening method for biliary atresia within YHC organizations.

## Materials and methods

### Design

We performed a qualitative study with semi-structured interviews performed in YHC organizations in the Netherlands. We followed the consolidated criteria for reporting qualitative research (COREQ) for designing the methodology of the study and in the reporting of the results. The Medical Ethics Review Board of the University Medical Center Groningen reviewed the study protocol and concluded that the Medical Research Involving Human Subjects Act was not applicable (METc UMCG 2023/149, Project ID 16779).

### Sample

We included Dutch YHC organizations of varying size from a national register, both organizations that implemented the ISCC, and organizations that did not. Per organization, we selected participants (YPH physicians and nurses) involved in the organizational policy regarding hyperbilirubinemia or were working with the ISCC in daily practice. We contacted a known YPH professional per organization to find out who was the appropriate interviewee within the organization (snowball sampling).

### Procedures and measures

**Fig. 2:**
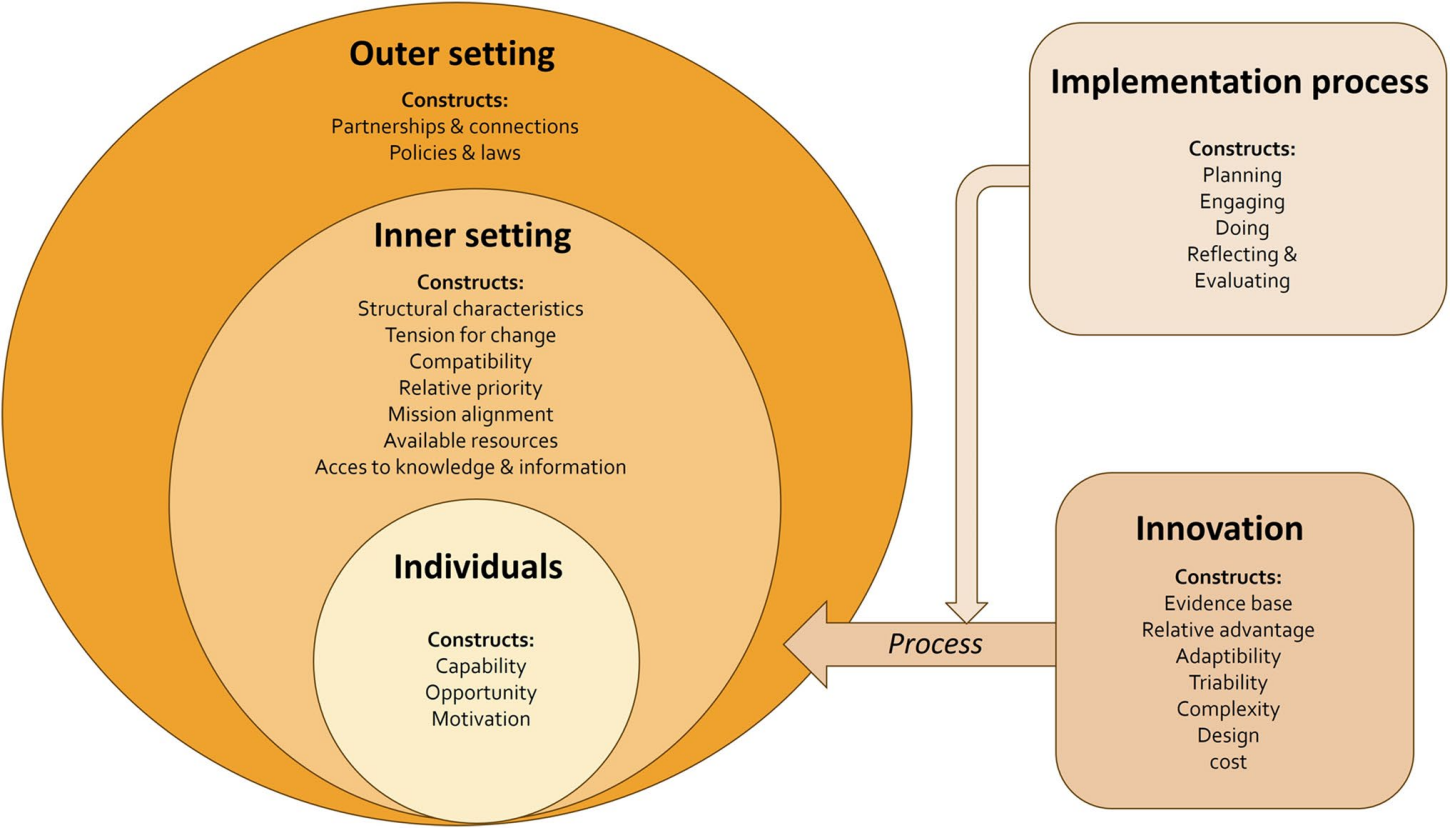
Schematic figure of the CFIR with the domains and construct used for designing the interview guide. Figure adapted from the CFIR 2.0 by Damschroder [16]

Each participant was interviewed online by the first author (LW, a YPH physician and experienced researcher), between October 2023 and February 2024. The first two interviews were performed with author LD. No topic list adjustments were needed. Before the interview, all participants received information about the aims and content of the study and signed an informed consent form. Semi-structured interviews were conducted, using a topic guide constructed from the Consolidated Framework of Implementation Research (CFIR) [16]. Following this framework, the interviews addressed five domains: innovation, inner setting, outer setting, individuals, and implementation process. In total, 23 of 48 CFIR constructs suited study aims and were used for developing questions for our interview guide (Fig. 2, Supplementary file 1).

The interviews lasted about 30–45 min and were recorded using Microsoft Teams. We conducted interviews until we reached saturation point (with a pre-determined minimum of seven YHC organizations) [17].

### Data handling and analysis

All interviews were transcribed verbatim by Microsoft Teams and were checked and anonymized by author LW. ATLAS.ti software was used for coding the transcripts. Four interviews were double coded by authors LW and LD. The code tree was discussed by the authors repeatedly; its final version is shown in Supplementary file 2. The CFIR was used to guide data coding and analysis. In the analysis, we first described the background of the sample. Next, we categorized codes per CFIR domain as pertaining to factors promoting or hindering implementation.

## Results

### Flow of the selection and characteristics of the sample

**Fig. 3:**
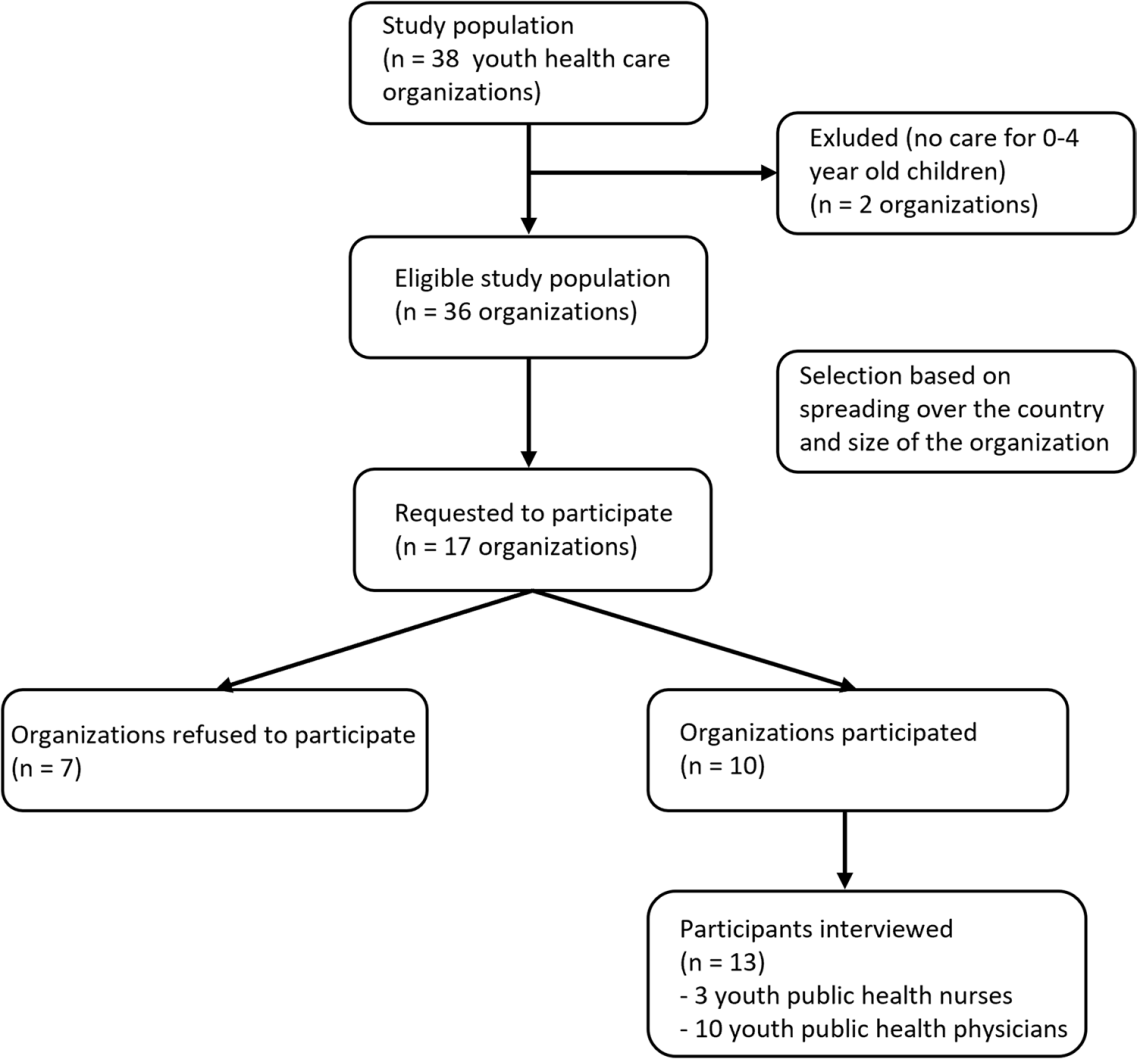
Flow diagram of participant selection

Ten of 17 approached YHC organizations participated, among which all three organizations that officially implemented the ISCC. Out of these 10 organizations, in total 10 physicians and 3 nurses were interviewed (Fig. 3). The reasons of non-participation were a lack of time of the responsible professional (*n* = 4) and lack of a responsible professional for the policy around hyperbilirubinemia (*n* = 3). Characteristics of the included sample are shown in Table 1.

**Table 1:** Characteristics of the sample (organizations and professionals)

	YHC organizations with ISCC	YHC organizations without ISCC
**Number**	3	7
**Size of organization**		
**- Small** (*covering an area* < *500,000 inhabitants)* **- Medium** *(covering an area 500,000–1 million inhabitants)* **- Large** *(covering an area* > *1 million inhabitants)*	0 2 1	3 3 1
**Professionals**		
**- YPH physicians** **- YPH nurses**	4 1	6 2
**Gender participants**		
**- Female** **- Male**	5 0	8 0

### Promoting and hindering factors

**Fig. 4:**
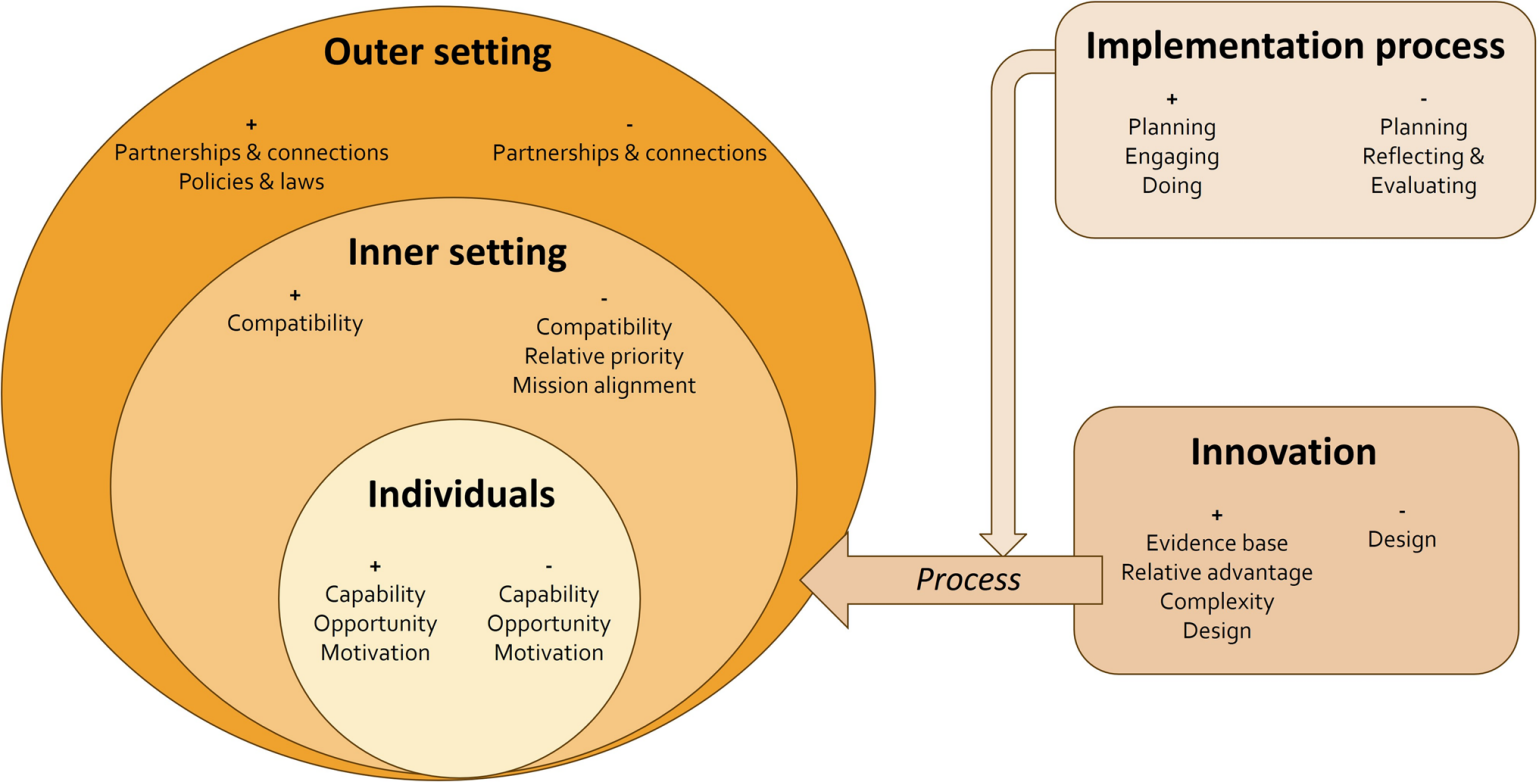
Summary of results of all organizations (*n* = 10) regarding hindering (-) and promoting (+) constructs per CFIR domain (see “Materials and methods” section)

Promoting and hindering factors are reported below per domain for organizations with and without implementation of the ISCC. An overview of these results is shown in Table 2 and Fig. 4. An overview of some quotes of participants is shown in Table 3.

**Table 2:** Overview of promoting and hindering factors for organizations that implemented the ISCC (*n* = 3) and organizations that did not (*n* = 7)

CFIR Domains	Promoting factors Organizations with ISCC	Promoting factors No implementation	Hindering factors Organizations with ISCC	Hindering factors No implementation
Individuals	- Positive attitude towards ISCC - Experience with the ISCC - Feel competent to use the ISCC - Difficulty in assessing jaundice by the professionals	- Positive attitude towards ISCC - Earlier experience with biliary atresia	- Deviation from guideline - Lack of knowledge - Lack of support - Lack of risk perception	- Deviation from guideline - Lack of knowledge - Lack of support for bilirubin test - High workload of professionals
Inner setting	- Good alignment with current workflow - Incorporation in digital patient record system	- Good alignment with current workflow	- Varying adherence to guideline between teams	- High workload during home visits - More interventions planned for implementation - First live visit to YHC organization > 4–6 weeks - Unclear policy on jaundice within organization - No responsible professional for organization policy on managing jaundice - Insufficient embedding of other YHC interventions
Outer setting	- Good collaboration with paediatricians - A standardized letter of referral to GP in case of prolonged jaundice - ISCC’s simplicity for parents	- Good collaboration with paediatricians - A standardized letter of referral to GP in case of prolonged jaundice - Inclusion of ISCC in national guideline	- Knowledge gap and lack of risk perception among GPs - Lack of awareness of the guideline among GPs - Lack of involvement of GPs at the start of the implementation	- Knowledge gap and lack of risk perception among GPs - Insufficient collaboration between YPH professionals, GPs and paediatricians on this topic
Process	- Good stakeholder engagement - ISCC documented in workflow with concise summary available - Appointing a contact person within each team - Appointing a YPH physician for ISCC-related questions - Interactive training - Involvement of an expert in the training for professionals	x	- Insufficient embedding of the intervention - Inadequate onboarding on ISCC - Lack of involvement of managers at the start of the implementation - Inability of YPH physicians to order lab tests directly	x
Innovation	- Minimal time commitment for professionals - Educates parents about stool assessment and reassures regarding normal stool colour variations - Minimal accompanying text on ISCC - Simplicity - Reinforces the crucial role of YHC in early disease detection - Integration within Dutch growth booklet	- Minimal time commitment for professionals - Educates parents about stool assessment and reassures regarding normal stool colour variations - Minimal accompanying text on ISCC - Simplicity - Supports professionals in early identification biliary atresia - Availability of a laminated card on the desk - Empowers parents - More medical tasks for nurses - Evidence based - Clinical topic	-	- Fear for colour shifting of the ISCC in the digital version

**Table 3:** Quotes of participants

Involving construct (domain)	Quote of participant
Quotes on promoting factors	
Compatibility (inner setting) and relative advantage (innovation)	“I think we’d like to implement that. It’s actually a relatively simple addition to the current protocol, and we see the value. Everyone is asking about those colours. However, it hasn’t been made very explicit with the colour chart yet. I think it’s fairly easy to implement”
Complexity (innovation)	“The YPH nurse states: ‘It’s such a useful tool and takes so little time, often only 2 min or even less. And this also applies to the four-week visit. It’s simply a question you ask: “What colour is the stool?” and you show the card and that’s it”
Compatibility (inner setting) and capability (individuals) and complexity (innovation)	“Yes, I think keeping it simple will work well. We’re already familiar with the guideline and this naturally fits in. It’s just a matter of refocusing our attention on it for a while, so I don’t think it requires major changes. A brief explanation within the team or a short training session should be sufficient”
Quotes on hindering factors	
Partnerships and connections (outer setting)	“The collaboration and handover processes between YPH physicians, GPs, and paediatricians could be enhanced for neonates with prolonged jaundice”
Capability (individuals)	“Nurses often notice if a baby is jaundiced at the two-week home visit, but they primarily focus on alertness, stool, urination, and feeding. If they consider the baby to be excessively jaundiced, they may refer them to the GP. However, it’s quite rare for them to take any action at that stage. We (YPH physicians) usually see the infants at four weeks, and it’s common to see prolonged jaundice in breastfed babies. Often, parents report that the jaundice is already improving. If not, I refer them to the GP at the four-week visit”
Reflecting and evaluating (implementation process)	“The implementation was completed some time ago, but unfortunately, in my opinion, many colleagues have already forgotten about it. It soon became apparent that not all teams are regularly ordering new copies of the card”
Capability (individuals)	“No, I don’t expect it to take a lot of extra time. It’s more, I think it needs to become a habit to do so. And I do wonder if people see the added value, because they will have to change their behaviour again. And that applies more to the nurses than the doctors. Because with jaundice, you always ask about the stool, and then you can consult the ISCC. For the nurses, it’s new, and I wonder if they have a good risk perception”

### Domain individuals

This domain refers to the roles and the characteristics of the participants.

#### Promoting factors

As major promoting factor, the YPH nurses and physicians who worked with the ISCC indicated that they felt competent to use the ISCC, because it is simple and takes little time (< 2 min). Participants had a positive attitude towards the ISCC independent of implementation status. Earlier professional experience with biliary atresia was reported as promoting factor at individual level to recognize the value of the addition of the ISCC to the workflow. Additionally, interviewees mentioned difficulties in recognizing jaundice by the professionals, which supports ISCC use.

#### Hindering factors

All participants reported that YPH professionals commonly and consciously deviate from the Hyperbilirubinemia guideline, which recommends bilirubin blood evaluation at 3 weeks for infants with prolonged jaundice. Main reasons for these deviations included the burden of an extra blood test and difficulties in interpreting the value of the bilirubin test due to the high incidence of breast milk jaundice. This lack of knowledge about biliary atresia and underestimation of the associated risks of a late diagnosis were reported by all organizations. Interviewees that did not use the ISCC expressed concern about an increased work load for YPH nurses due to an additional task during home visits.

### Domain inner setting

This domain refers to the setting in which the innovation is implemented, in this study the participating YHC organizations.

#### Promoting factors

All participants agreed that early biliary atresia detection aligns with existing YHC responsibilities. Participants that already worked with the ISCC said the integration seamlessly blends with the workflow within their organization, which includes a 2-week home visit by a YPH nurse and a 4-week consultation with a YPH physician. These interviewees also highlighted the effectiveness of incorporating the ISCC into the digital patient record system, because it streamlined the process and ensures readily accessible ISCC information.

#### Hindering factors

Organizations that implemented the ISCC mentioned variation in ISCC utilization between YPH teams, with lack of repeated training as one of the reasons. In line with this, organizations without implementation reported inadequate embedding of other YHC interventions due to lack of repeated training as a risk for implementation of the ISCC. Some interviewees also reported that their organization policy about jaundice is unclear. Another hindering factor for implementation was the lack of an officially assigned responsible person for the policy on managing jaundice within the organization (*n* = 4). Further, the already high workload for YPH nurses during 2-week home visits was mentioned as barrier. One organization did not perform a in-person visit before the age of 4 weeks with all infants, which makes early introduction of the ISCC more difficult.

A couple of organizations reported that several other interventions for YPH professionals are planned for implementation, which could influence the timing of ISCC implementation.

### Domain outer setting

This domain refers to the context of the outer setting. In this study, it regards GPs, paediatricians, parents, and the Association of Dutch Youth Health Care Physicians.

#### Promoting factors

Strong collaboration with paediatricians was reported to be a facilitator for successful ISCC implementation, as it would lead to a shared approach. Interviewees working with the ISCC noted that a standard referral letter with clear instructions about the bilirubin test and interpretation for GPs aided the referral process for prolonged jaundice. Participants reported that parents found the ISCC user-friendly. Organizations that did not implement the ISCC suggested that inclusion of the ISCC in the national guideline would promote implementation.

#### Hindering factors

All participants reported a lack of knowledge and risk perception among GPs as a barrier to implementing a screening based on the ISCC. As GPs are responsible for ordering bilirubin tests, they play a crucial role in early biliary atresia diagnosis. Interviewees reported that GPs often attributed prolonged jaundice to breastfeeding and were less familiar with the current multidisciplinary guideline on hyperbilirubinemia. All participants identified insufficient collaboration with GPs on this topic as a potential cause. The organizations implementing the ISCC recommended involving GPs from the beginning, which was not done initially.

### Domain implementation process

This domain refers to the activities and strategies used to implement the innovation.

#### Promoting factors

Participants emphasized the importance of providing comprehensive interactive training and education on the ISCC’s rationale and application, involving experts. Engaging key stakeholders within organizations (professionals, screeners, policy advisors, managers, the customer contact centre) was considered beneficial. Additionally, appointing a contact person within each local YPH team and a YPH physician for ISCC-related questions was seen as helpful. Providing a concise summery of the ISCC workflow within the teams was considered beneficial.

#### Hindering factors

Insufficient embedding of the implementation of the ISCC was identified as a major barrier. Trainings were not repeated after the initial implementation phase, and the onboarding of new professionals regarding the ISCC was not always adequate, despite the clearly documented workflow. The lack of involvement of managers from the outset of implementation was also perceived as a barrier, as they play a crucial role in managing the team’s tasks. The inability of YPH professionals to directly order lab tests was seen as a barrier to the overall biliary atresia detection process.

### Domain innovation

This domain refers to the innovation itself, i.e. the ISCC.

#### Promoting factors

Participants viewed the ISCC as a clinically relevant intervention that contributes to early biliary atresia detection. They praised its simplicity for both professionals and parents, as well as the minimal time commitment required from professionals. Another facilitator was the user-friendly design of the ISCC, which could be integrated into the Dutch growth booklet with minimal accompanying text. This is particularly advantageous for non-native speakers. In addition, participants viewed the card as a valuable tool for educating parents about stool assessment and providing reassurance about normal stool colour variations. Moreover, the ISCC emphasizes the crucial role and the expertise of YPH professionals in early disease detection.

#### Hindering factors

Organizations that did not implement the ISCC mentioned the trend towards digitalisation within their organizations as a potential barrier to ISCC implementation, because the risk of colour shifting.

## Discussion

This study explored factors that promote and hinder the implementation of the ISCC within YHC organizations. Key promoting factors included positive attitudes towards the ISCC, a sense of professional competence, the ISCC’s simplicity, compatibility with workflows, and the involvement of key stakeholders in the implementation process. Major hindering factors comprised limited knowledge and inadequate risk perception of biliary atresia among YPH professionals and GPs, insufficient ISCC embedding within YHC organizations, and inadequate interprofessional collaboration with GPs.

### Professional level

At the level of professionals, positive attitudes and sense of competence were positively influenced by the ISCC’s simplicity and minimal time commitment. This is consistent with previous findings that good feasibility of interventions supports implementation [18–20]. Also, consistent with the findings of Witt et al. [5], professionals valued the ISCC, a valuable tool for educating and reassuring parents about normal stool colour variations.

A primary barrier at professional level was a lack of knowledge and inadequate risk perception on biliary atresia, which likely contributes to current deviations from guideline recommendations. This is in line with Francke et al. [18] who showed that effective guideline adoption depends on healthcare professionals’ comprehension of the clinical presentation and the underlying rationale for the guideline. Furthermore, as shown by Vincenten et al. [19], professional biases, such as an overemphasis on breastmilk jaundice, can significantly hinder the implementation process. Consequently, improving professionals’ knowledge and risk perception of biliary atresia is crucial for successful ISCC implementation.

### Organizational level

At organizational level, compatibility with existing workflows emerged as promoting factor for the implementation. This is consistent with prior research that emphasized the importance of workflow alignment for successful adoption of new interventions [18, 20, 21]. The concern of increased work load expressed by YHC organizations without implementation was contradicted by organizations that implemented the ISCC, who praised the simplicity and the minimal time commitment of the ISCC.

Major organizational barriers to implementation were the insufficient embedding of the ISCC within YHC organizations and insufficient collaboration with GPs. This confirms previous finding of limited integration of interventions into organizational structures and processes being a barrier to implementation [20, 21]. The negative effect of insufficient collaboration with GPs confirms findings of Ploeg et al. [21], on the importance of inter-organizational collaborations for successful implementation. Rogers et al. [22] also described the hindering effect of absence of a designated professional and reported leadership engagement as an important facilitator for successful implementation. To ensure sustained implementation of the ISCC, organizations should appoint a dedicated individual to oversee the process and provide comprehensive, interactive training for all healthcare professionals, and collaborate with GP associations.

### Strengths and limitations

This study possesses several strengths. It included a diverse range of YHC organizations, varying in size, implementation status, and geographical distribution. Moreover, it encompassed perspectives from both YPH physicians and nurses, both crucial for ISCC implementation. Additionally, the inclusion of professionals involved in policymaking and clinical practice provided valuable insights.

Some limitations should be acknowledged as well. First, we may have missed overloaded professionals, which would mean that our findings are somewhat too positive on potential for implementation. However, the identified organizational barriers, such as the need for improved embedding and a designated responsible individual, suggest a degree of critical self-reflection within the participating organizations. Second, the interviewer being a YPH physician could have reinforced socially desirable responding. This impact is probably limited by mitigating measures, such as guaranteed confidentiality. This seems to have been effective, given that some participants reported guideline deviation.

### Implications for practice and research

The main hindering factors provide opportunities for enhanced ISCC implementation. In particular at the level of professionals, knowledge and risk perception of YPH professionals and GPs about biliary atresia should be improved. Participants emphasized the need for comprehensive, interactive training and education on the ISCC’s rationale and application. This training should involve experts and colleagues with experience in biliary atresia and ISCC use. The recently launched ‘Early detection and diagnosis of biliary atresia’ guideline [15] is a valuable resource for professionals, as it explicates the importance of biliary atresia compared to previous versions and explicitly advises YPH professionals to utilize the ISCC and refer cases with discoloured stools directly to a paediatrician or GP. Better knowledge and risk perception of biliary atresia are likely to enhance guideline adherence.

Also at organizational level, several factors require attention to ensure sustained implementation of the ISCC. Organizations should appoint a dedicated professional for the hyperbilirubinemia policy, who could oversee the implementation process. This dedicated professional could make working agreements with regional GPs and paediatricians, based on the recently launched guideline [15]. Furthermore, this professional could guarantee repeated comprehensive, interactive trainings for all healthcare professionals, including new employees, enhancing the knowledge and risk awareness of professionals.

Furthermore, the cost-effectiveness of the ISCC in universal screening for biliary atresia in the Netherlands remains to be confirmed. It should be noted though that ample evidence from large-scale studies in various countries suggests that use of the ISCC leads to earlier biliary atresia detection and, consequently, improved patient outcomes [5, 10–13].

## Supplementary Information

Below is the link to the electronic supplementary material.

Supplementary file1 (TIFF 6637 KB)

Supplementary file2 (DOCX 27 KB)

## Data Availability

Data is provided within the manuscript or supplementary information files. Original interviews are stored at the electronic database of the Dept. of Health Sciences of the University Medical Center Groningen.

## Abbreviations

CFIR: Consolidated framework for implementation research
COREQ: Criteria for reporting qualitative research
GPs: General practitioners
ISCC: Infant Stool Colour Card
YPH: Youth public health
YHC: Youth health care
