# Supplementary figures and images for: Promoting and hindering factors for implementation of the Infant Stool Colour Card in Dutch youth health care organizations
Source: Eur J Pediatr. 2025 Jun 4;184(7):390. doi: 10.1007/s00431-025-06212-7 (PMC12137367; doi:10.1007/s00431-025-06212-7)

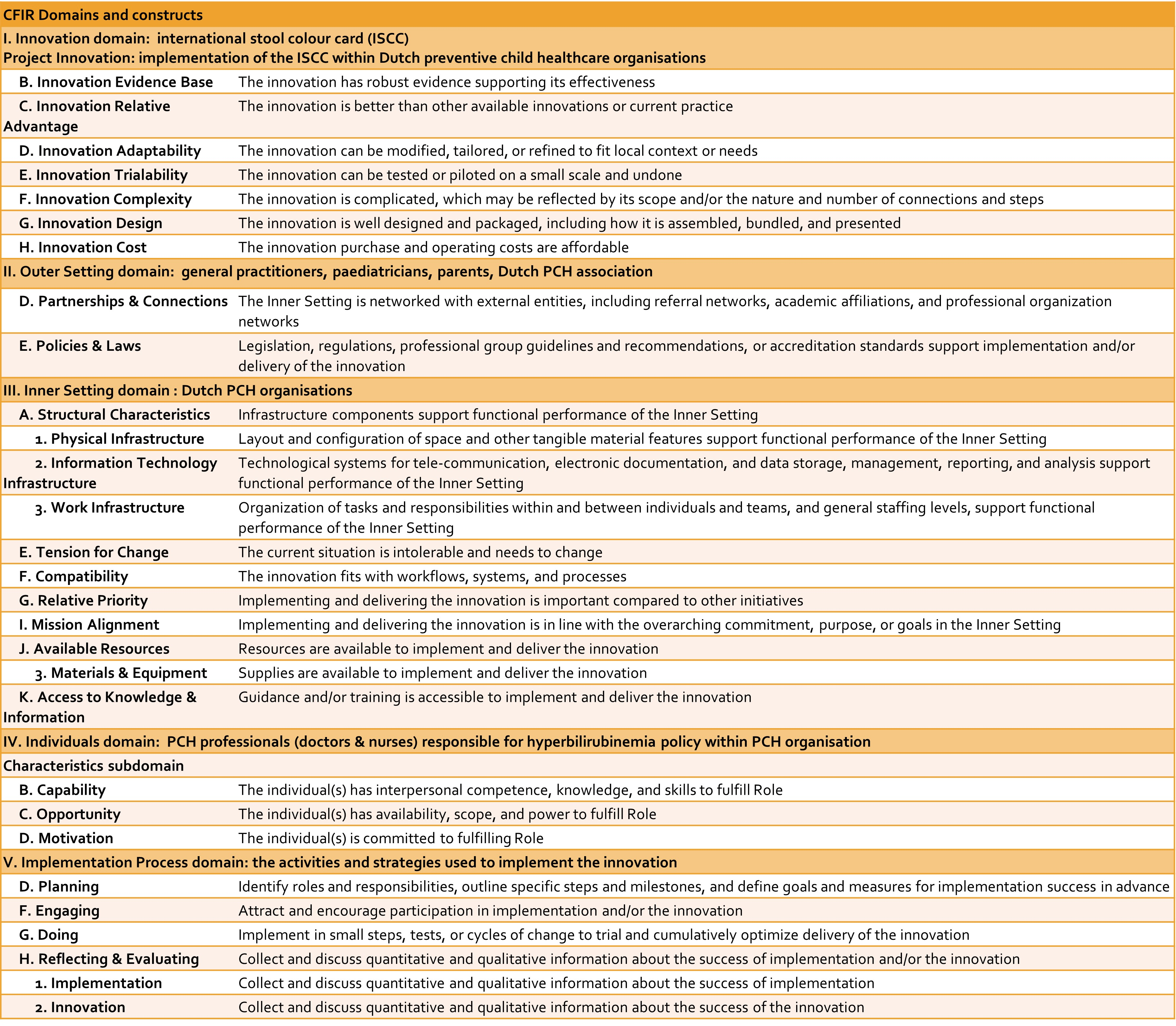

Supplement: Supplementary file 1 — Supplementary file1 (TIFF 6637 KB) [file 431_2025_6212_MOESM1_ESM.tiff]
