# Supplementary material for: Promoting and hindering factors for implementation of the Infant Stool Colour Card in Dutch youth health care organizations
Source: Eur J Pediatr. 2025 Jun 4;184(7):390. doi: 10.1007/s00431-025-06212-7 (PMC12137367; doi:10.1007/s00431-025-06212-7)
Supplement: Supplementary file 2 — Supplementary file2 (DOCX 27 KB) [file 431_2025_6212_MOESM2_ESM.docx]

**Supplementary file 2: code tree**

| **Code tree** | | | |
| --- | --- | --- | --- |
| ○ General |  |  |  |
|  | ○ position of interviewed professional |  |  |
| ○ no implementation |  |  |  |
|  | ○ inner setting |  |  |
|  |  | ○ ISCC fits in workflow | |
|  |  | ○ priority ISCC | |
|  |  | ○ adherence to hyperbilirubinemia guideline | |
|  |  | ○ expected added value of pilot | |
|  |  | ○ vision of current guideline | |
|  |  | ○ vision of current workflow | |
|  | ○ intervention characteristics |  |  |
|  |  | ○ promoting factors of implementation | |
|  |  | ○ ease of use by professional | |
|  |  | gebruiksgemak professional- | ○ duration explanation ISCC to parents |
|  |  | gebruiksgemak professional- | ○ explanation ISCC to parents |
|  |  | ○ expected hindering factors ISCC | |
|  |  | ○ expected promoting factors ISCC | |
|  |  | ○ design ISCC | |
|  | ○ characteristics professional |  |  |
|  |  | ○ own case with biliairy atresia | |
|  |  | ○ personal attitude towards ISCC | |
|  |  | ○ personal opinion about guideline | |
|  |  | ○ personal believe succesrate implementation | |
|  | ○ outer setting |  |  |
|  |  | ○ user friendliness parent | |
|  |  | gebruiksgemak ouder- | ○ interpretation colors |
|  |  | ○ attitude parent towards ISCC | |
|  |  | ○ collaboration laboratory | |
|  |  | ○ collaboration GP | |
|  |  | samenwerking huisarts- | ○ lack of knowledge |
|  |  | samenwerking huisarts- | ○ working agreements |
|  |  | ○ collaboration pediatrician | |
|  | ○ proces implementation |  |  |
|  |  | ○ supplies implementation | |
|  |  | benodigdheden implementatie | ○ communication parents |
|  |  | benodigdheden implementatie | ○ digital patient record |
|  |  | benodigdheden implementatie | ○ experiences of organisations already implemented ISCC |
|  |  | benodigdheden implementatie | ○ expert |
|  |  | benodigdheden implementatie | ○ materials |
|  |  | benodigdheden implementatie | ○ education |
|  |  | benodigdheden implementatie | ○ simplicity |
|  |  | benodigdheden implementatie | ○ scientific evidence and knowledge |
|  |  | ○ implementation method: pilot | |
|  |  | ○ possible barrier | |
|  |  | mogelijke barrières | ○ embedding |
|  |  | mogelijke barrières | ○ staff formation |
|  |  | mogelijke barrières | ○ attitude parent towards ISCC |
|  |  | mogelijke barrières | ○ collaboration GP and pediatrician |
|  |  | mogelijke barrières | ○ education |
|  |  | mogelijke barrières | ○ time professionals |
|  |  | mogelijke barrières | ○ responsibility policy |
|  |  | ○ key figures | |
| ○ ISCC implemented |  |  |  |
|  | ○ inner setting |  |  |
|  |  | ○ available resources | |
|  |  |  | ○ manpower |
|  |  |  | ○ time |
|  |  | ○ ISCC workflow | |
|  |  |  | ○ experiences |
|  |  |  | ○ ISCC fits in workflow |
|  |  |  | ○ variation utilisation within teams |
|  |  |  | ○ workflow ISCC |
|  |  | ○ guideline hyperbilinemia | |
|  |  |  | ○ adjustment workflow |
|  |  |  | ○ barrier parent |
|  |  |  | ○ current workflow jaundice |
|  |  |  | ○ adherence to guideline |
|  | ○ intervention characteristics |  |  |
|  |  | ○ accompanying text ISCC | |
|  |  | ○ involvement digital patient record | |
|  |  | ○ experienced benefits ISCC | |
|  |  | ○ experiences pilot phase | |
|  |  | ○ ease of use by professional | |
|  |  | gebruiksgemak professional | ○ interpretation colors |
|  |  | gebruiksgemak professional | ○ duration explanation |
|  |  | gebruiksgemak professional | ○ explanation ISCC |
|  |  | ○ costs implementation ISCC | |
|  |  | ○ design ISCC | |
|  | ○ characteristics of professional |  |  |
|  |  | ○ competence use ISCC | |
|  |  | ○ attitude participant towards ISCC | |
|  |  | ○ attitude professional towards guideline | |
|  |  | ○ knowledge professionals | |
|  | ○ outer setting |  |  |
|  |  | ○ message for other organisations | |
|  |  | ○ ease of use by parents | |
|  |  | gebruiksgemak ouder | ○ barrier |
|  |  | gebruiksgemak ouder | ○ accompanying text |
|  |  | gebruiksgemak ouder | ○ use of ISCC |
|  |  | gebruiksgemak ouder | ○ interpretation colors by parent |
|  |  | gebruiksgemak ouder | ○ design ISCC parent |
|  |  | ○ collaboration with GP | |
|  |  |  | ○ general |
|  |  |  | ○ communication |
|  |  |  | ○ knowledge |
|  |  |  | ○ risk perception |
|  |  | ○ collaboration with pediatrician | |
|  |  | ○ collaboration with laboratory | |
|  | ○ proces implementation |  |  |
|  |  | ○ designated professional | |
|  |  | ○ cause implementation | |
|  |  | ○ adjustments implementation | |
|  |  | ○ barriers implementation | |
|  |  | ○ promoting factors implementation | |
|  |  | ○ embedding | |
|  |  | ○ evaluation | |
|  |  | ○ implementation steps in general | |
|  |  | implementatie stappen algemeen | ○ implementation steps |
|  |  | implementatie stappen algemeen | ○ work plan |
|  |  | implementatie stappen algemeen | ○ Client contact center |
|  |  | implementatie stappen algemeen | ○ pilotfase |
|  |  | implementatie stappen algemeen | ○ education |
|  |  | ○ key figures | |
|  |  | ○ implementation phase | |
|  |  | stadium implementatie | ○ differences between team-implementation |
|  |  | ○ convincing steps for professionals | |
|  |  | ○ improvement points for implementation | |
|  |  | verbeterpunten implementatie | ○ embedding |
|  |  | verbeterpunten implementatie | ○ digital ISCC-card |
|  |  | verbeterpunten implementatie | ○ support professionals |
|  |  | verbeterpunten implementatie | ○ evaluation |
|  |  | verbeterpunten implementatie | ○ knowledge professionals |
|  |  | verbeterpunten implementatie | ○ risk perception professionals |
|  |  | verbeterpunten implementatie | ○ role managers |
|  |  | verbeterpunten implementatie | ○ collaboriation GP |
|  |  | verbeterpunten implementatie | ○ collaboration pediatrician |
